# The growth patterns of the medical technology profession in South Africa

**DOI:** 10.4102/ajlm.v10i1.1164

**Published:** 2021-04-23

**Authors:** Malcolm T. Ellapen, Terry J. Ellapen, Yvonne Paul

**Affiliations:** 1 Department of Virology, Albert Luthuli Hospital, Durban, South Africa; 2 Department of Sport, Rehabilitation and Dental Sciences, Faculty of Health, Tshwane University of Technology, Pretoria, South Africa

**Keywords:** Medical technology, attrition, growth, interns, practitioner

## Abstract

**Background:**

Constant appraisal of healthcare workforce trends is vital; this measure determines the adequacy of the workforce in meeting its society’s healthcare demands. This includes determining the number of the incoming workforce (students, interns) and the active or practising workforce relevant registries.

**Objective:**

This study aimed to examine patterns of workforce growth in the medical technology profession (students, interns and practitioners) from 2008 to 2018 in South Africa.

**Methods:**

Student, intern and practitioner medical technology registries, from the 2012/2013, 2016/2017 and 2017/2018 Health Professions Council of South Africa (HPCSA) annual reports, were analysed. The number of National Health Laboratory Service and private laboratory posts were secured from the National Health Laboratory Service plan performance report. A comparison between the total number of South African medical technology (private and public) posts occupied versus the HPCSA practitioner 2016 register was completed, to determine the saturation status.

**Results:**

Annual student, intern, and practitioner registries indicated a mean growth of 6.8%, 28.9%, and 0.7% from 2008 to 2018. The transition of interns to practitioners is progressively dwindling (2015–2018). The practitioner register showed a 1.2% decline in registration from 2013 until 2018. In 2016, only 55.9% of the HPCSA registered medical technologists were employed (*p* < 0.001).

**Conclusion:**

There are more medical technologists than available public and private sector posts. The progressively growing student register compared to the dwindling practitioner register indicates attrition in the profession. An investigation identifying the reasons why graduates neglect to register as practising medical technologists should be undertaken.

## Introduction

Sub-Saharan African countries have fewer employed healthcare staff compared to Australia, the United Kingdom and the United States.^1^ In 2013, these developed countries had an estimated 59.4 healthcare practitioners per 10 000 of their populations, compared to an estimated 22.8 healthcare practitioners per 10 000 obtainable in sub-Saharan African countries.^1,2^ This deficiency in the healthcare staff to population ratio adversely influences the quality of healthcare provision in sub-Saharan African countries.

Lopes et al. described the attrition in a profession as the premature voluntary resignation of an employee from their vocation due to illness, death, emigration or expected superannuation.^3^ Common reasons cited for premature exit from healthcare professions are poor salary packages, transition to more lucrative employment opportunities, job dissatisfaction, lack of upward job mobility, poor working conditions and family responsibilities.^2,4^ The primary reason for job dissatisfaction is unrealistic employment expectations.^5^ In many healthcare professions, interns are expected to register as practising healthcare professionals immediately after completing their mandatory residency in order to secure employment.^6^ A new scenario is, however, emerging where graduate laboratory scientists refrain from registering and practising as healthcare scientists or technologists after completing their residency.^2,7,8^ This contributes to the poor profession transition which adversely affects the pool of available scientists.^2^ Most research pertaining to career transition on *brain drain* has concentrated on doctors, professional nurses and pharmacists who prematurely leave their professions.^2,9,10^ A literature search on the Sabinet search engine (January to July 2019), employing the keywords ‘*attrition of medical technologists*’ revealed 15 records. Following title and abstract analyses, only a single study relating to this research interest remained, which is suggestive of the paucity of literature on the aforementioned topic in Africa.^2^ Anecdotal reports also suggest that the South African medical technology profession may be experiencing attrition.

Medical technologists analyse human tissue, as well as body fluids or excretions; laboratory findings are then utilised by the medical practitioner or dentist to make a clinical diagnosis or institute medical or dental treatment.^6^ The constant scarcity of medical technologists and laboratory scientists in the United Kingdom and the United States inspired research aimed at uncovering the reasons for their premature attrition from the medical profession. Data have informed the development and implementation of some laboratory staff retention strategies.^11,12,13^ Despite the important role that medical technologists play concerning clinical diagnoses, there is little research on this workforce’s growth in South Africa. This preliminary investigation aimed to identify the medical technology profession’s growth patterns by reviewing the student, intern and practitioner medical technology registries from 2008 to 2018 on the Health Professions Council of South Africa (HPCSA) database. It is intended that the preliminary findings of this article will encourage further research to uncover the predisposing factors influencing the intern medical technologists’ attrition that will in turn inform retention strategies.

## Methods

### Ethical considerations

These data are in the public domain (HPCSA website); therefore, no ethical approval for the study was needed.

### Study design and data source

The study was a retrospective descriptive analysis of the medical technology profession growth in South Africa. The period observed was from 2008 to 2018. The student, intern and practitioner registries of the medical technology profession were obtained from the HPCSA annual reports.^14,15,16^ A student medical technologist-in-training is currently completing a medical technology degree and is registered with the HPCSA as a student-in-training. An intern medical technologist is a student who has completed their theoretical requirements and has partially fulfilled the practical requirements for the medical technology degree but is currently in the final year of mandatory clinical residency. Being a student, the intern medical technologist is therefore registered with the HPCSA. A practitioner is a medical technologist who has completed the theoretical requirements for the academic degree and the mandatory clinical residency of both the training institution and the HPCSA. The HPCSA register of medical technologists serves to identify all eligible practitioners. Without HPCSA registration a medical technologist cannot practise.

### Statistical analysis

The percentage change in registration between each successive year among the students, interns and practitioners was calculated as shown in Equation 1^17^:

$$\%\text{ growth}=\left(\frac{\text{Present year registration}-\text{previous registration}}{\text{present year registration}}\right)\times 100$$ [Eqn 1]

To determine the growth or attrition of the medical technology professionals in a given year, the number of registered interns was compared to the number of newly registered practitioners in the subsequent year. Growth or attrition was calculated as:

$$\%\text{ difference}=\left(\frac{\text{present year NRP}-\text{previous year IMT}}{\text{present year NRP}}\right)\times 100$$ [Eqn 2]

In Equation 2, NRP is the number of newly registered practitioners and IMT is the number of intern medical technologists.

The comparison of the total number of medical technologists employed in 2016 according to the Competition Commission Health Market Inquiry versus the number of HPCSA registered medical technologists was made to determine existence of over-saturation or under-saturation in the profession.^14,15,18^ The descriptive statistics adopted were mean, standard deviation and percentages. A chi-square analysis, between the numbers of National Health Laboratory Service and private laboratory posts in comparison to the number of practitioners, was completed for the year 2016. An independent student t-test was used to compare annual intern to practitioner medical technologist transition rate, as well as the annual growth rate of interns and practitioners (alpha was set at *p* < 0.05).

## Results

Overall, there was a positive increase in the number of student, intern and practitioner medical technologists (Table 1).^14,15^ The mean growth in the number of student medical technologists was 6.8% while that of the interns was 28.9%, despite observed declines in 2015 and 2018. The annual registration of practitioners showed a mean growth of 0.7% over the period of 2008 to 2018 (Table 1).^14,15^ Table 2 shows the intern to practitioner rate; an increase was recorded from 2008 to 2012, while from 2013 to 2018 the number of interns registering as practitioners following their residency dwindled. The annual practitioner registry growth rate from 2008 until 2012 was 2.3%, as compared to the diminishing growth rate of −1.2% from 2013 until 2018. A comparative review of the growth of the intern medical technologists and practitioner registries from 2013 until 2018 significantly differed (*p* = 0.04). Intern medical technologist registries grew at an annual mean rate of 12.7% (±15.3), while practitioner registries dwindled by an annual mean rate of −1.2% (±6.7%). Table 3 indicates a significant difference between the number of newly registered interns compared to the registered practitioners (*p* < 0.047).

**TABLE 1:** Growth rate patterns of student, intern and practitioner registries of the South African medical technology profession (2008–2018).

Year	Student-in-training		Intern		Practitioner	
	*n*	% growth	*n*	% growth	*n*	% growth
2008	2556	-	1	-	5151	-
2009	2925	12.61	1	0.00	5311	3.01
2010	3367	13.12	57	98.24	5383	1.33
2011	3624	7.09	146	60.95	5552	3.04
2012	3734	2.94	321	54.51	5761	3.62
2013	Unavailable	Unavailable	453	29.13	5794	0.56
2014	3882	-	549	17.48	5075	−14.16
2015	4213	7.85	519	−5.78	5257	3.46
2016	4335	2.81	630	17.61	5362	1.95
2017	4265	−1.64	835	24.55	5480	2.15
2018	4725	9.73	782	−6.77	5637	2.78
Mean % growth	-	6.8	-	28.9	-	0.7

*Source*: HPCSA annual reports. Health Professions Council of South Africa. Annual report (2012/2013). Pretoria: HPCSA. 2013; p. 21. Health Professions Council of South Africa. Annual report (2017/2018). Pretoria: HPCSA.

**TABLE 2:** The transition rate of change of South African medical technologists interns to practitioners (2008–2018).

Year	IMT	Practitioners	NRP	% difference between NRP and IMT
2008	1	5151	103	-
2009	1	5311	160	99.37
2010	57	5383	72	98.61
2011	146	5552	169	66.27
2012	321	5761	209	30.14
2013	453	5794	33	−872.72
2014	549	5075	−719	−163.0
2015	519	5257	182	−201.64
2016	630	5362	105	−394.28
2017	835	5480	157	−433.89
2018	453	5637	-	−431.84
Mean	360.4 (±278.1)	5433 (±261.3)	53.54 (±236.0)	−220.29

IMT, interns medical technologist; NRP, newly registered practitioners.

**TABLE 3:** Comparison between the transitional number of South African interns, practitioners and newly registered practitioners from 2008 to 2018.

Type of professional	Previously registered	Newly registered	*t*-test
Mean number of interns	360.4 (±278.1)	53.54 (±236.0)	0.01
Mean number of practitioners	5433 (±261.3)	53.54 (±236.0)	0.0001

There are both public and private pathological laboratories and depots in South Africa. The public laboratories and depots fall within the ambit of the South African National Health Laboratory Service. The largest South African private pathological laboratories include PathCare, Ampath and Lancet; these saturate approximately 90% of the private market.^18^ In 2016, the Competition Commission Health Market Inquiry reported that the South African National Pathology Group had a total number of 10 295 employees, which comprised 3000 medical technologists. Of the 3000 medical technologists, National Health Laboratory Service employed 1473 practitioners (49.1%).^18^ Table 3 indicates that the number of previously registered practitioners significantly differed from the newly registered practitioners (*p* < 0.0001).

## Discussion

The HPCSA student register is composed of students pursuing a vocation of medical technology, who are registered with one of the accredited South African tertiary training institutions. Students-in-training cannot complete clinical practice, if they are not registered with the HPCSA.^6^ However the student register does not distinguish among first-year, second-year and third-year students-in-training. The register does distinguish student-in-training, intern medical technologist and practitioner.^6^ The student register increased from 2008 to 2018. These statistics are reassuring, and they epitomise the ongoing interest in the medical technology profession. Anagnostopoulou et al. and Ossai et al. reported that undergraduates who are pleased with the quality of education and residency they receive continue with the academic process.^19,20^ As such the steady growth in the student registration can be seen as arising out of the satisfaction of both students and their benefactors with the quality of education and training provided by South African tertiary education institutions. However, to ascertain whether the growth in the student registry is a direct result of the satisfaction that students and their benefactors feel, regarding the quality of their training and education, a more direct investigation of the perceptions of students-in-training should be undertaken.

The progression of student-in-training medical technologists to intern medical technologists is marked by positive average growth. This is reminiscent of the fact that undergraduates are ardent to earn their degree and these statistics furthermore reflect the work-integrated learning stage of the clinical internship, which falls under the purview of both academic training institutions and clinical laboratories. Clinical internships are offered by both public and private laboratories, allowing students the opportunity to complete their clinical training.

The practitioner register showed a meagre positive growth rate from 2008 to 2018 of 0.7%. This rate is below the global growth rate of the medical technology profession which has fluctuated between 2% and 8% from 2010 to 2018, with an average growth rate of 3.65%.^21^ In 2016 the HPCSA had 5362 registered medical technologists (Table 2), but according to the Competition Commission Health Market Inquiry only 3000 (55.9%) were employed in both public and private healthcare sectors; 2362 (44.1%) were unemployed or not practising.^14,15,18^ This statistic shows that there were 2362 medical technologists unaccounted for, which could be seen as over-saturation of the profession.

There was a steady decline in the intern to practitioner transition rate from 2013 to 2018, stressing the attrition of intern medical technologists to practitioners. These findings concur with those of Noden et al. who reported that many intern and graduate medical technologists are actively leaving the profession while some seek further postgraduate education.^2^ The precise rationale for exist from the South African medical technology profession is unknown as yet. Therefore, further investigation is warranted. While there are several possible reasons for this premature exodus from the profession, McClure postulated that the primary reason many healthcare laboratory graduates and interns do not practise the profession is due to poor career advancement opportunities. Thus, interns are upskilling themselves to be more marketable in other alternative, more financially lucrative, professions.^7^ The annual medical technologist salary packages offered by the various South African pathological laboratories range as follows: Ampath (South African Rand [ZAR] R247 000.00), Lancet Laboratories (R249 000.00), National Health Laboratory Service (R287 000.00) and PathCare (R314 000.00).^22^ The United States annual salary packages for medical technologists range from $53 576.00 United States dollars (USD) to $52 712.00 USD, which is between R931 150.88 to R916 134.56 (conversion rate of $1.00 USD:R17.38 on 02 June 2020 [using conversion rate http://www.payscale.com]). In Hong Kong, the average medical technologist salary package is Hong Kong dollars (HKD) $264 906.00, which is equivalent to R593 389.44 (conversion rate of $1.00 HKD:R2.24 on 02 June 2020). A common alternative career path is academia. The average annual South African academic salary is R309 281.00, which is almost equivalent to the highest salary package in PathCare.^21^ Beck and Doig reported that poor financial remuneration packages are fundamental stimuli for premature graduate and young healthcare laboratory staff resignation.^12^ Another reason is that many older medical scientists continue working until retirement age, thereby limiting the employment opportunities of graduates.^8^ Young graduates are thus driven to change their professions to secure employment. Presently there are more registered medical technologists than there are available practitioner posts in both the private and public sectors.^17^ The initial problem the authors identified from the preliminary data is that there is a lack of jobs for approximately 40% of the practitioner registry; where are these practitioners going to find an occupation? This information needs to be relayed to the training institutions to reduce the student entrance numbers, which can help prevent further over-saturation. A pragmatic solution would be to create more medical technologist posts, because most laboratories are understaffed, resulting in the staff being overworked.

### Recommendations for prospective studies

The study confirms the attrition of the profession at the point of a career transition from intern medical technologist to practitioner. The possible reasons for this remain unknown. It is recommended that an investigation identifying the reasons why graduates neglect to register as practising medical technologists be undertaken. The attrition of healthcare laboratory staff in developed countries has encouraged intensive research, which uncovered the rationale behind their decisions.^11,12,13^ It is suggested that similar investigations be undertaken in South Africa. The study should also try to determine the factors predisposing the change in student-in-training, intern medical technologist and practitioner perceptions and attitudes from initial desire to pursue a career in medical technology to leaving the profession.

### Limitations

This preliminary study has the following limitations, which warrant further investigation: how many intern medical technologists complete their residency, what are the potential career options of those interns who complete their residency, how many interns who complete their residency continue to stay in South Africa, how many travel aboard to secure jobs and how many leave the profession?

### Conclusion

The South African medical technology profession has steadily grown its student and intern medical technologist registers. The evidence indicates that there are more registered practitioners available than public and private sector posts (suggesting potential over-saturation). Also, many intern medical technologists are refraining from registering as practitioners, concurring with anecdotal reports of the ongoing attrition of the profession in South Africa. It is postulated that the lack of employment opportunities in South Africa is driving the attrition of intern to practitioner medical technologist transition. The concern lies in a not-so-distant future scenario, where there may be under-saturation of medical technologists in South Africa, created by the present attrition of intern to practitioner transition. However, the underlying factors predisposing this attrition need to be determined in prospective investigations. Future investigations determining the perceptions and attitudes of students-in-training, intern medical technologists and practitioners towards continuing in the profession is essential. The evidence gleaned from these investigations should be employed by the medical technology profession in conjunction with the South African Department of Health to draft and implement strategies to prevent attrition and over-saturation.
